# Carcinoembryonic Antigen (CEA)-Specific 4-1BB-Costimulation Induced by CEA-Targeted 4-1BB-Agonistic Trimerbodies

**DOI:** 10.3389/fimmu.2019.01791

**Published:** 2019-07-31

**Authors:** Kasper Mikkelsen, Seandean Lykke Harwood, Marta Compte, Nekane Merino, Kasper Mølgaard, Simon Lykkemark, Ana Alvarez-Mendez, Francisco J. Blanco, Luis Álvarez-Vallina

**Affiliations:** ^1^Immunotherapy and Cell Engineering Laboratory, Department of Engineering, Aarhus University, Aarhus, Denmark; ^2^Department of Antibody Engineering, Leadartis SL, Madrid, Spain; ^3^Structural Biology Unit, CIC bioGUNE, Parque Tecnológico de Bizkaia, Derio, Spain; ^4^Department of Nursing, Universidad Complutense de Madrid, Madrid, Spain; ^5^IKERBASQUE, Basque Foundation for Science, Bilbao, Spain; ^6^Cancer Immunotherapy Unit (UNICA), Department of Immunology, Hospital Universitario 12 de Octubre, Madrid, Spain; ^7^Immuno-Oncology and Immunotherapy Group, Instituto de Investigación Sanitaria 12 de Octubre (i+12), Madrid, Spain

**Keywords:** cancer immunotherapy, immunostimulatory antibodies, costimulation, 4-1BB, 4-1BB agonists, trimerbodies, CEA, CEA-targeted 4-1BB agonists

## Abstract

4-1BB (CD137) is an inducible costimulatory receptor that promotes expansion and survival of activated T cells; and IgG-based 4-1BB-agonistic monoclonal antibodies exhibited potent antitumor activity in clinical trials. However, the clinical development of those antibodies is restricted by major off-tumor toxicities associated with FcγR interactions. We have recently generated an EGFR-targeted 4-1BB-agonistic trimerbody that demonstrated strong antitumor activity and did not induce systemic inflammatory cytokine secretion and hepatotoxicity associated with first-generation 4-1BB agonists. Here, we generate a bispecific 4-1BB-agonistic trimerbody targeting the carcinoembryonic antigen (CEA) that is highly expressed in cancers of diverse origins. The CEA-targeted anti-4-1BB-agonistic trimerbody consists of three 4-1BB-specific single-chain fragment variable antibodies and three anti-CEA single-domain antibodies positioned around a murine collagen XVIII-derived homotrimerization domain. The trimerbody was produced as a homogenous, non-aggregating, soluble protein purifiable by standard affinity chromatographic methods. The purified trimerbody was found to be trimeric in solution, very efficient at recognizing 4-1BB and CEA, and potently costimulating T cells *in vitro* in the presence of CEA. Therefore, trimerbody-based tumor-targeted 4-1BB costimulation is a broadly applicable and clinically feasible approach to enhance the costimulatory environment of disseminated tumor lesions.

## Introduction

In the last decade, immunostimulatory monoclonal antibodies (mAbs) have risen to prominence as a clinically viable approach to induce a tumor-specific immune response in cancer patients (1). The antagonistic mAbs used in immune checkpoint blockade are able to block T cell-inhibitory signaling from receptors such as cytotoxic T-lymphocyte-associated antigen 4 (CTLA-4) and programmed cell death-1 (PD-1), and have been successfully used in the treatment of several types of cancers (1). Another type of cancer immunotherapy uses agonistic mAbs to achieve the same effect, but from the opposite direction (2).The agonism of T cell costimulatory receptors can enhance T cell effector functions, proliferation, and survival, which has been demonstrated using several receptors from the tumor necrosis factor (TNF) receptor superfamily (TNFRSF). One such TNFRSF member, 4-1BB (CD137, TNFRSF9) (3), forms the basis for the immunostimulatory approach used in this article.

In addition to activated T cells, 4-1BB is expressed on dendritic cells, NK cells, B cells, and endothelial cells. It is only expressed and present on T cells for a short interval occurring after TCR engagement, and has a single identified ligand, 4-1BBL, found on antigen presenting cells (4). The engagement of 4-1BB by its ligand or agonistic mAbs provides substantial boosts to the T cell response (4), which prompted the incorporation of 4-1BB intracellular signaling domain into TCR-like chimeric antigen receptors (CARs) and ultimately greatly improved their functionality (5).

Melero et al. (6) were the first to report that anti-4-1BB-agonistic mAbs induce regression of established tumors in mice. The long-lasting tumor-specific immune response was primarily mediated by CD8+ T cells (6). Two anti-human 4-1BB-agonistic mAbs have been generated and are undergoing clinical evaluation. Urelumab (BMS-663513) is a non-ligand–blocking, fully human IgG_4_ mAb with an engineered hinge region to improve stability. A monotherapy urelumab trial was associated with serious hepatic toxicity that resulted in two deaths, leading to its interruption (7). Utomilumab (PF-05082566) is a ligand-blocking, fully human IgG_2_ mAb with a better safety profile than urelumab, but with only modest antitumor activity (3).

Therefore, and considering that toxicity has been the major impediment for IgG-based anti-4-1BB-agonistic mAbs, and that different studies suggest that organ toxicities are dependent on FcγR interactions, new costimulatory strategies that are not based on full-length antibodies, are urgently needed. Targeted strategies confining the costimulatory activity to the tumor site appears as attractive approaches to improve the implementation of immunomodulatory molecules (8, 9). We have recently generated a tumor-targeted 4-1BB-agonistic trimerbody, consisting of three anti-4-1BB single-chain fragment variable (scFv) antibodies, and three anti-EGFR single-domain (V_HH_) antibodies positioned in an hexagonal conformation around a murine collagen XVIII-derived homotrimerization (TIE^XVIII^) domain (10). The Fc-free trimerbody exhibited potent antitumor activity, and did not induce systemic cytokine secretion and hepatotoxicity associated with first-generation 4-1BB-agonistic mAbs (10).

EGFR is widely expressed in normal, non-neoplastic tissues, and its use as a target antigen can lead to severe on-target, off-tumor immunotoxicity. For this reason, we have investigated the targeting of carcinoembryonic antigen (CEA) by a bispecific 4-1BB-agonistic trimerbody. CEA is a heavily glycosylated protein that belongs to the CEA-related cell adhesion molecule (CEACAM) family of the immunoglobulin gene superfamily, and is highly expressed in cancers of diverse origins, especially carcinomas (11).

The CEA-targeted 4-1BB-agonistic trimerbody was produced as a homogenous, non-aggregating, soluble protein purifiable by standard affinity chromatographic methods. The purified trimerbody was found to be trimeric in solution, very efficient at recognizing 4-1BB and CEA, and potently costimulated T cells *in vitro* in the presence of CEA.

## Materials and Methods

### Mice

C57BL/6 female mice were purchased from Envigo Crs (Barcelona, Spain), and were maintained in controlled environment: temperature (21 ± 1°C), humidity (50 ± 5%), and 12 h light/dark cycles. Manipulation was performed under aseptic conditions in a laminar flow hood, when was necessary, and sterilized water and food were available *ad libitum*. All animal procedures conformed to European Union Directive 86/609/EEC and Recommendation 2007/526/EC, enforced in Spanish law under RD 1201/2005. Animal protocols were approved by the Ethic Committee of Animal Experimentation of the Instituto Investigación Sanitaria Puerta de Hierro-Segovia de Arana (Hospital Universitario Puerta de Hierro Majadahonda, Madrid, Spain). Procedures were additionally approved by the Animal Welfare Division of the Environmental Affairs Council of the Government of Madrid (66/14).

### Cells and Culture Conditions

HEK293 cells (CRL-1573) were from the American Type Culture Collection (Rockville, MD, USA) and were cultured in Dulbecco's modified Eagle's medium (DMEM) (Lonza, Walkersville, MD, USA) supplemented with 2 mM L-glutamine, 10% (vol/vol) heat inactivated fetal calf serum (FCS) and antibiotics (all from Life Technologies, Carlsbad, CA, USA) referred as to DMEM complete medium (DCM). HEK293 cells expressing m4-1BB (HEK239^m4−1BB^) were provided by Dr I. Melero (CIMA, Navarra, Spain) (12) and were cultured in DCM + 500 μg/ml G418 (Sigma-Aldrich, St. Louis, MO, USA). Cells were routinely tested for mycoplasma by PCR using the Mycoplasma Plus TM Primer Set (Stratagene, Cedar Creek, TX, USA).

### Construction of Expression Vectors

The mammalian expression vector pCR3.1-1D8^N18^encoding the 4-1BB-specific 1D8 scFv-based N-terminal trimerbody has been previously described (10). To construct the plasmid pCR3.1-1D8^N5^TIE^C18^CEA.1, a synthetic gene encoding the C-terminal part of the 1D8 scFv gene fused by a 5-mer linker to the N-terminus of the human TIE^XVIII^ domain a by 18-mer linker to the CEA-specific CEA.1 V_HH_ gene (13) was synthesized by Geneart AG (Regensburg, Germany) and subcloned as *Pst*I/*Xba*I into the vector pCR3.1-1D8^N18^. The vector pCR3.1-CEA.1-TIE^17^ encoding the CEA-specific CEA.1 V^HH^-based N-terminal trimerbody CEA.1^N17^ has been previously described (14). The sequences were verified using primers FwCMV (5′-CGCAAATGGGCGG TAGGCGTG-3′) and RvBGH (5′-TAGAA GGCACAGTCGAGG-3).

### Expression and Purification of Recombinant Antibodies

HEK293 cells were transfected with the appropriate expression vectors using Lipofectamine 2,000 (Life Technologies) according to the manufacturer's protocol and selected in DCM with 500 μg/ml G418 to generate stable cell lines. Conditioned media from transiently and stably transfected cells were analyzed by ELISA, western blotting and FACS. Recombinant antibodies were purified from conditioned media with HisTrap HP columns (GE Healthcare, Uppsala, Sweden).

### Western Blotting

Samples were separated under reducing conditions on 12% Tris-glycine gels and transferred to a nitrocellulose membrane using iBlot 2 Dry Blotting System (Life Technologies). The membrane was blocked with Odyssey Blocking Buffer (LI-COR Biosciences, Lincoln, NE, USA) and probed with anti-c-myc mAb (clone 9E10, Abcam, Cambridge, UK), followed by incubation with DyLight800-goat anti-mouse (GAM) IgG (Rockland Immunochemicals, Gilbertsville, PA, USA).Visualization of protein bands was performed with the Odyssey^®^ system (LI-COR Biosciences).

### ELISA

The ability of trimerbodies to bind purified antigens was studied by ELISA as described (10, 14). Briefly, Maxisorp (NUNC Brand Products, Roskilde, Denmark) plates were coated (2 μg/well) with recombinant human CEACAM5/CD66e (hCEA; Sino Biological, Beijing, P.R.China) or recombinant mouse 4-1BB:human Fc chimera (m4-1BB; R&D Systems, Minneapolis, MN, USA). After washing and blocking with 300 μl 5% BSA in PBS, 100 μl of neat conditioned media from transfected HEK293 cells or purified protein solution (1 μg/ml), were added and incubated for 1 h at room temperature. After three washes, 100 μl of HRP-conjugated goat anti-mouse IgG (Sigma-Aldrich) were added for 1 h at room temperature, after which the plate was washed and developed. To demonstrate the simultaneous binding of the two antibody domains present in the bispecific trimerbody a dual ELISA was performed. Briefly, Maxisorp plates were coated with hCEA and after washing and blocking, 100 μl of neat supernatant from transfected HEK293 cells, were added and incubated for 1 h at room temperature. After washing, 100 μl of m4-1BB (2 μg/ml) were added followed by 100 μl of HRP-conjugated goat anti-human-Fc IgG (Sigma-Aldrich) and incubated for 1 h at room temperature. The plate was washed and developed.

### Flow Cytometry

The ability of trimerbodies to bind to cell surface 4-1BB was studied by flow cytometry as described previously (10). Briefly, HEK293 or HEK293^4−1BB^ cells were incubated with supernatants or purified trimerbodies (5 μg/ml) and anti-c-myc mAb for 30 min. After washing, the cells were treated with appropriate dilutions of PE-conjugated goat F(ab')_2_ anti-mouse IgG (Jackson Immuno Research, Newmarket, UK). The samples were analyzed with a SonySH800 (Sony, Tokyo, Japan). The mouse anti-human HLA-ABC mAb (clone W6/32, Abcam) was used as a control in the flow cytometry studies.

### Circular Dichroism (CD)

Circular dichroism measurements were performed with a Jasco J-810 spectropolarimeter (JASCO). The spectrum was recorded on a protein sample at 2.8 μM in PBS using a 0.2 cm path length quartz cuvette at 25°C. Thermal denaturation curve from 7 to 95°C was recorded on the same protein sample and cuvette by increasing temperature at a rate of 1°C/min and measuring the change in ellipticity at 218 nm.

### Size Exclusion Chromatography-Multiangle Light Scattering (SEC-MALS)

Static light scattering experiments were performed at room temperature using a Superdex 200 Increase 10/300 GL column (GE Healthcare) attached in-line to a DAWN-HELEOS light scattering detector and an Optilab rEX differential refractive index detector (Wyatt Technology Corporation, Santa Barbara, CA, USA). The column was equilibrated with running buffer (PBS, 0.1 μm filtered) and the SEC-MALS system was calibrated with a sample of BSA at 1 g/L in the same buffer. Then a 100 μL sample of the antibody at 0.3 g/L in PBS was injected into the column at a flow rate of 0.5 mL/min. Data acquisition and analysis were performed using ASTRA software (Wyatt Technology Corporation). Based on numerous measurements on BSA samples at 1 g/L under the same or similar conditions we estimate that the experimental error in the molar mass is around 5%.

### Mass Spectrometry

A 10 μl protein sample was desalted using ZipTip^®^ C4 micro-columns (Merck Millipore, Burlington, MS, USA) and eluted with 0.5 μl SA [sinapinic acid, 10 mg/ml in [70:30] Acetonitrile: Trifluoroacetic acid 0.1%] matrix onto a GroundSteel massive 384 target (Bruker Daltonics, Billerica, MA, USA). An Autoflex III MALDI-TOF/TOF spectrometer (Bruker Daltonics) was used in linear mode with the following settings: 5,000–40,000 Th window, linear positive mode, ion source 1: 20 kV, ion source 2: 18.5 kV, lens: 9 kV, pulsed ion extraction of 120 ns, high gating ion suppression up to 1,000 Mr. Mass calibration was performed externally with protein 1 standard calibration mixture (Bruker Daltonics). Data acquisition, peak peaking and subsequent spectra analysis was performed using FlexControl 3.0 and FlexAnalysis 3.0 software (Bruker Daltonics).

### Kinetics and Binding Studies Using Biolayer Interferometry

The ability of 1D8^N/C^CEA.1 to bind biosensor-immobilized m4-1BB and hCEA in solution at the same time was investigated using biolayer interferometry (BLI) on an Octet RED96 system (Fortebio, Menlo Park, CA, USA). AR2G biosensors (Fortebio) were activated using s-NHS and EDC, and then coated with 10 μg/mL m4-1BB in 10 mM sodium acetate at pH 6 for 20 min, followed by quenching with ethanolamine. After 10 min of equilibration in kinetics buffer (PBS with 0.1% BSA and 0.05% Tween20), the coated biosensors were incubated in kinetics buffer containing 4 nM of 1D8^N18^, 1D8^N/C^CEA.1, or only kinetics buffer for 1 h. The biosensors were then transferred into a solution of 50 nM hCEA in kinetics buffer or kinetics buffer for 2 h, followed by a final incubation in kinetics buffer for 1 h. Raw sensor data was exported and smoothed by Savitky-Golay filtering. Similarly, the kinetics of the interaction between m4-1BB and the trimerbody were investigated by using m4-1BB-coated biosensors, incubating with 2 or 4 nM of 1D8^N/C^CEA.1 in kinetics buffer for 1 h, and then monitoring dissociation in kinetics buffer for 3 h. The data were fit to a 1:1 binding model using the Octet Analysis software.

### Cell Adhesion Assay

96-well microtiter plates (Corning Costar, Cambridge, MA, USA) were coated overnight at 4 °C with (2 μg/well) hCEA or (1 μg/well) laminin-111 (Lm111) extracted from the Engelbreth-Holm-Swarm mouse tumor (Sigma-Aldrich). After washing and blocking with 200 μl 3% BSA-DMEM for 1 h at 37°C, appropriated dilutions of purified 1D8^N18^ or 1D8^N/C^CEA.1 were added for 1 h at 4°C. After washing 5 × 10^4^ HEK293 or HEK239^m4−1BB^ cells were loaded per well in serum-free medium and incubated for 30 min in humidified 5% CO_2_ atmosphere at 37°C. After washing 100 μl of substrate CellTiter-Glo (Promega, Madison, WI, USA) were added per well, and the bioluminescence measured using a Tecan Infinite F200 plate-reading luminometer (Tecan Group Ltd., Zurich, Switzerland). Results are expressed as a mean ± SD (*n* = 3) from 1 of at least 3 separate experiments. Data are reported as the fold change in adhesion relative to BSA.

### Antigen-Specific T Cell Costimulation Assays

Goat anti-hamster IgG (Jackson ImmunoResearch) and hCEA were pre-coated (5 μg/ml) overnight at 4°C in 96-well plates. After blocking, 1 μg/ml anti-CD3ε mAb (clone 145-2C11; Immunostep, Salamanca, Spain) was added and incubated at 37°C for 1 h. Purified CD8a+ T cells (CD8a+T Cell Isolation Kit, mouse, Miltenyi Biotec, GmbH) from spleens of C57BL/6 mice were added (2 × 10^5^/well) in complete RPMI + 50 μM 2-mercaptoethanol with purified antibodies at 6.67 nM. As a control, purified mouse CD8a^+^ T cells were cultured alone with the immobilized anti-CD3ε mAb. After 72 h, cell proliferation was assessed using the CellTiter-Glo luminescent assay, and supernatants were collected and assayed for IFNγ secretion by ELISA (Diaclone, Besançon, France). Results are expressed as a mean ± SD (*n* = *3*) from 1 of at least 3 separate experiments.

### Statistical Analysis

All the experiments were done in triplicates and the statistical analysis was performed using Prism software (GraphPad Software, San Diego, CA, USA). Significant differences (*P* value) were discriminated by applying a two-tailed, unpaired Student's *t* test assuming a normal distribution with ^*^P < 0.05, ^**^P < 0.01, ^***^P < 0.001. Values are presented as mean ± SD.

## Results

### Design and Expression of a CEA-Targeted 4-1BB-Agonistic Trimerbody

**Figure 1:**
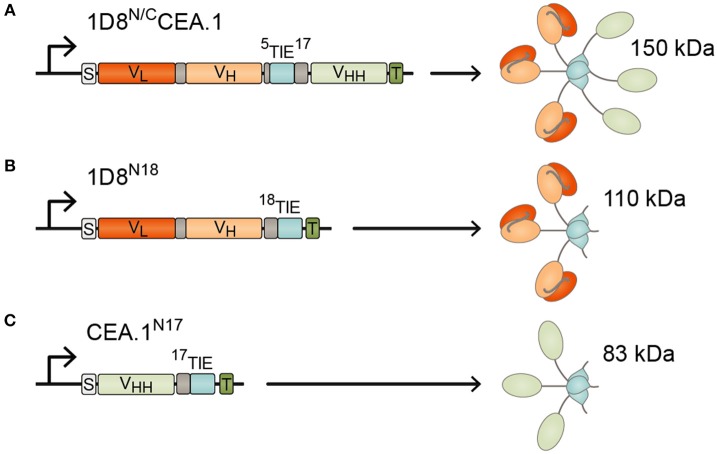
Schematic diagrams showing the genetic (left) and domain structure (right) of the bispecific 1D8^N/C^CEA.1 trimerbody **(A)**, and the monospecific 1D8^N18^ **(B)** and CEA.1^N17^ **(C)** trimerbodies. The variable regions (V_L_-V_H_) derived from 1D8 antibody are represented in red/orange, the anti-CEA V_HH_ CEA.1 in light green, the TIE^XVIII^ domains in light blue, and the linker regions in gray. All the trimerbodies contain a signal peptide from oncostatin M (white box), and a His6-myc tag (dark green box). Arrows indicate the direction of transcription.

**Figure 2:**
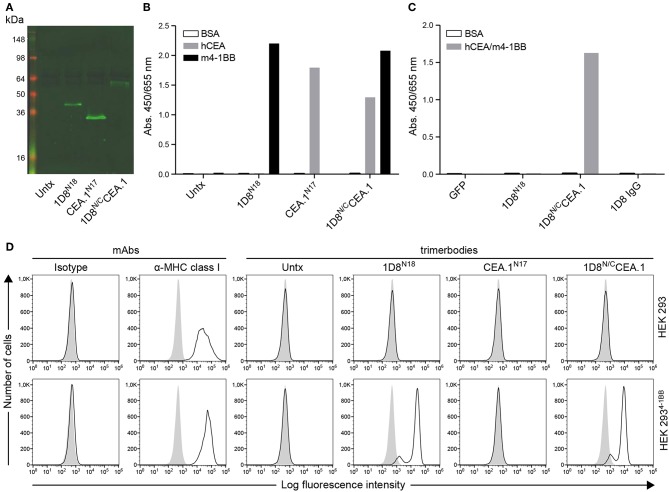
Characterization of secreted 1D8^N/C^CEA.1trimerbody. **(A)** The presence of secreted trimerbodies in the conditioned media from untransfected (Untx) or transfected HEK293 cells was demonstrated by western blot analysis. Migration distances of molecular mass markers are indicated (kDa). The blots were developed with anti-c-myc mAb, followed by incubation with an IRDye800-conjugated goat anti-mouse IgG. The functionality of secreted trimerbodies was demonstrated by ELISA against plastic immobilized hCEA and m4-1BB **(B)**. Simultaneous binding to hCEA and m4-1BB was assessed by dual ELISA by direct immobilization of hCEA, followed by 100 μl of neat supernatant from transfected HEK293 cells and addition of m4-1BB **(C)**. Flow cytometry on HEK293 cells and HEK293^m4−1BB^ cells **(D)**, using 100 μl of neat supernatant from transiently transfected HEK293 cells. An anti-MHC class I mAb was used as control.

To generate the CEA-targeted 4-1BB-agonist we fused the anti-human CEA V_HH_ (CEA.1) (13) to the C-terminus of 1D8^N5^ (10) through a 17-residues linker giving the 1D8^N/C^CEA.1 trimerbody (Figure 1A). The 1D8^N5^ is a compact scFv-based N-terminal trimerbody in which the 1D8 scFv is connected to the murine collagen XVIII-derived homotrimerization (TIE^XVIII^) domain with a 5-residue-long linker (10). The construct was designed with a c-myc and His tag at the C-terminus of the CEA.1 V_HH_. The construct was secreted by transfected HEK293 cells at similar levels to 1D8^N18^ but at lower levels than the V_HH_-based N-terminal trimerbody CEA.1^N17^ (Figure 2A). 1D8^N18^ is a scFv-based N-terminal trimerbody with an 18-residue linker (Figure 1B) (10), and CEA.1^N17^ is V_HH_-based N-terminal trimerbody with a 17-residue-long linker (Figure 1C) (14). ELISA analysis demonstrated that 1D8^N18^ specifically recognized mouse 4-1BB Fc chimera (m4-1BB), CEA.1^N17^ specifically recognized human CEA (hCEA), whereas 1D8^N/C^CEA.1 showed specific binding to both antigens (Figure 2B). Furthermore, when conditioned medium from 1D8^N/C^CEA.1-transfected HEK293 cells was added to hCEA-coated wells and, the CEA-bound trimerbodies were able to capture soluble m4-1BB (Figure 2C). The ability to interact with cell surface expressed m4-1BB was studied by flow cytometry. 1D8^N18^ and 1D8^N/C^CEA.1 bound to HEK293 cells transfected to express mouse 4-1BB on their cell surface (HEK293^m4−1BB^), but not to untransfected HEK293 cells. No binding was detected for CEA.1^N17^ (Figure 2D).

### Structural Characterization of the CEA-Targeted 4-1BB-Agonistic Trimerbody

**Figure 3:**
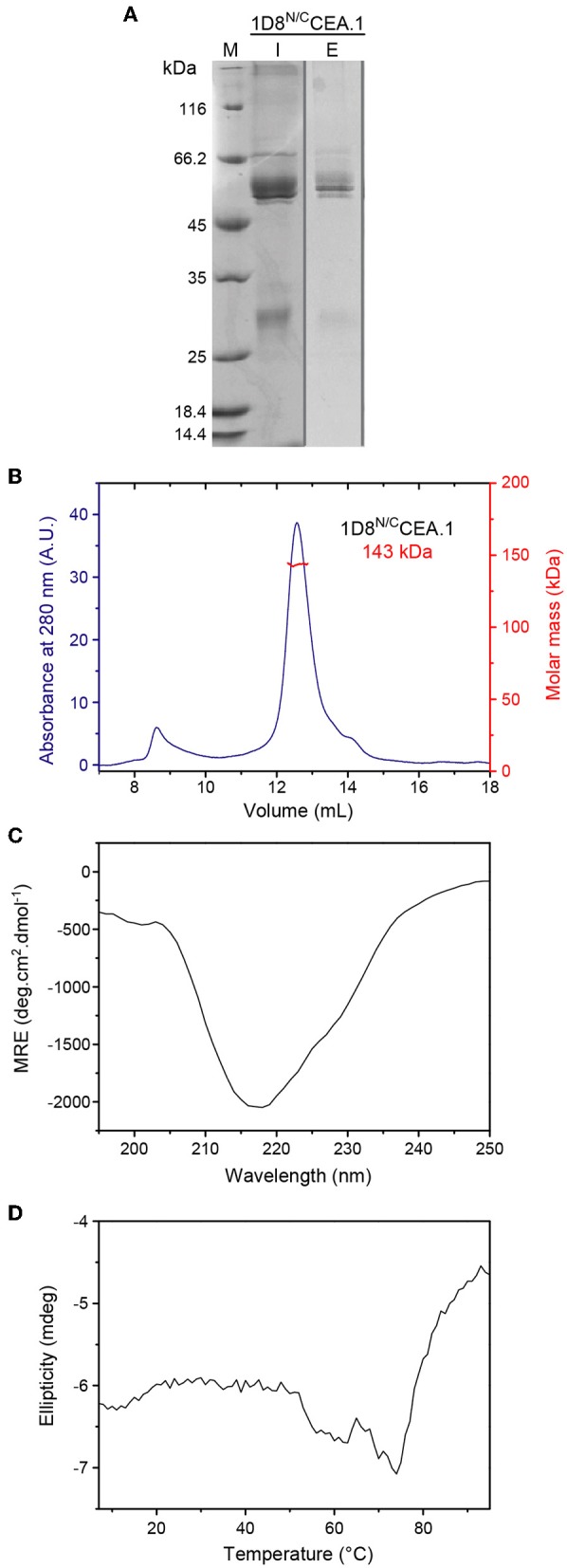
Structural characterization of purified 1D8^N/C^CEA.1 trimerbody. **(A)** Reducing SDS-PAGE of 1D8^N/C^CEA.1. The sample injected in the SEC column (I, 5 μL of the 0.3 g/L stock) and eluted at 12.6 mL (E; total protein present in the central 300 μL fraction was precipitated before loading). The gray vertical line indicates that the lanes belong to different gels. **(B)** Oligomeric analysis of the purified 1D8^N/C^CEA.1 trimerbody by SEC-MALS with the indicated molecular mass measured at the center of the chromatography peak. **(C)** Circular dichroism spectrum (mean residue ellipticity) of 1D8^N/C^CEA.1, and **(D)** thermal denaturation measured by the change in ellipticity at 218 nm.

The 1D8^N/C^CEA.1 was produced in stably transfected HEK293 cells and purified from conditioned medium by immobilized metal affinity chromatography, which yielded proteins with a high degree of purity as determined by reducing SDS-PAGE (Figure 3A). Mass spectrometry indicates a molecular weight of 50.4 kDa, which is the expected weight for the protomer after signal sequence processing (data not shown). The SEC-MALS chromatogram (Figure 3B) shows a major peak eluting at 12.6 mL with a molar mass of 143 kDa at the center of the peak, which indicates the formation of trimers in solution (with a calculated mass of 151.2 kDa). The chromatography also shows a minor proportion of aggregated material eluting at 8.7 mL, the exclusion volume of the column. The circular dichroism spectrum, with a minimum at 218 nm is consistent with a predominantly β-sheet secondary structure, which is stable up to ~50°C (Figures 3C,D). The thermal denaturation is irreversible as a large pellet was observed in the cuvette.

### Functional Characterization of the CEA-Targeted 4-1BB-Agonistic Trimerbody

**Figure 4:**
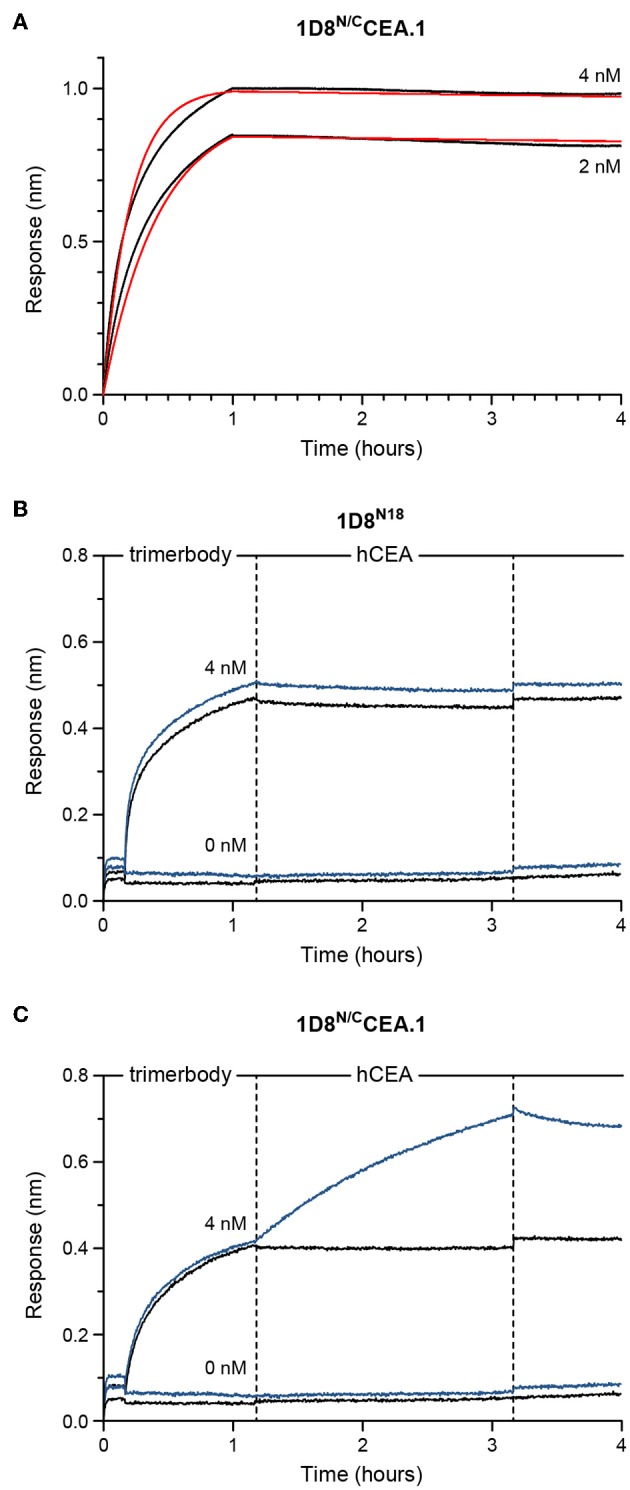
Functional characterization of purified 1D8^N/C^CEA.1 trimerbody using biolayer interferometry (BLI). **(A)** Experimental responses (black traces) and fitting to a 1:1 binding model (red traces) for 4 or 2 nM, as labeled, of 1D8^N/C^CEA.1 interacting with m4-1BB immobilized onto biosensors. **(B,C)** Experimental responses when either 1D8^N18^ **(B)** or 1D8^N/C^CEA.1 **(C)** were first allowed to interact with immobilized m4-1BB at concentrations of 4 or 0 nM, as labeled. These biosensors were then transferred into 50 nM of hCEA in solution (blue traces) or kinetics buffer without hCEA (black traces). 1D8^N/C^CEA.1 binds to both antigens, while 1D8^N18^ binds m4-1BB; hCEA did not interact with the m4-1BB-coated biosensors in the absence of trimerbody.

The binding kinetics of the bispecific 1D8^N/C^CEA.1 was investigated by biolayer interferometry (BLI) using biosensors coated with m4-1BB (Figure 4A). As shown in Figure 4A, the 1D8^N/C^CEA.1 showed saturating binding to m4-1BB at low nanomolar concentrations and an extremely slow dissociation (with an interaction half-life >40 h, as <5% dissociation was observed over 3 h), which is consistent with the binding behavior previously reported for m4-1BB-binding trimerbodies (10). We also used BLI to demonstrate the simultaneous binding of 1D8^N/C^CEA.1 to both m4-1BB and hCEA. 1D8^N/C^CEA.1 and 1D8^N18^ were both able to associate with sensor-immobilized m4-1BB, and 1D8^N/C^CEA.1 showed a subsequent binding to hCEA in solution (Figures 4B,C). Importantly, this indicates that the binding to either antigen does not sterically inhibit binding to the other antigen, which would prevent 1D8^N/C^CEA.1 from participating in the crosslinking of T cells and tumor cells.

**Figure 5:**
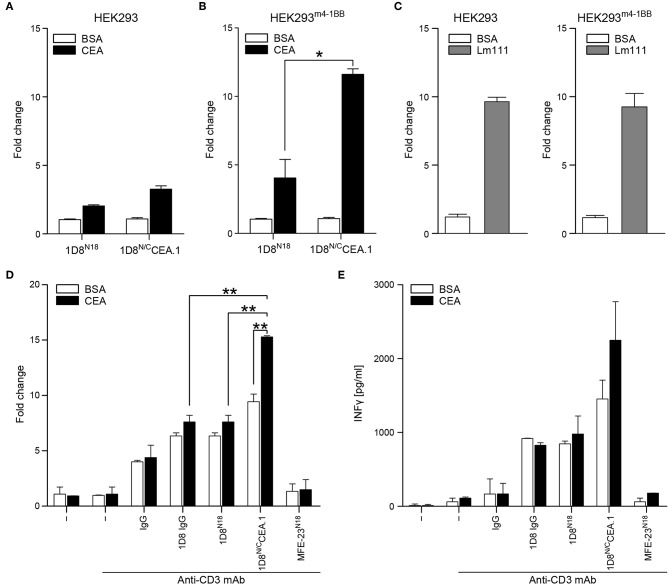
Functional characterization of 1D8^N/C^CEA.1 trimerbody. Adhesion of HEK293 cells **(A)** and HEK293^m4−1BB^cells **(B)** to plastic-immobilized BSA or CEA, after incubation with purified 1D8^N18^ or 1D8^N/C^CEA.1 trimerbodies. Adhesion of HEK293 cells and HEK293^m4−1BB^ cells to plastic-immobilized laminin 111 (Lm111) was used as a control **(C)**. Data are plotted as the fold change in adhesion relative to BSA. Costimulatory activity of anti-4-1BB antibodies **(D,E)**. Mouse CD8a+ T cells were plated with immobilized anti-CD3 mAb and hCEA or BSA in the presence of 1D8 IgG, 1D8^N18^, or 1D8^N/C^CEA.1, and proliferation **(D)** and IFN-γ secretion **(E)** were determined after 48 h. All the results are representative of one of three independent experiments. Data are mean ± SD (*n* = 3), *P < 0.05, **P ≤ 0.01, Student's *t*-test.

To further assess the multivalence and multispecificity of the 1D8^N/C^CEA.1 trimerbody, we performed cell adhesion assays to plastic immobilized hCEA or laminin (Lm111), as positive control. As shown in Figure 5B, HEK293^m4−1BB^ cells adhered to wells coated with hCEA after incubation with 1D8^N/C^CEA.1. Moreover, 1D8^N/C^CEA.1 was as efficient as Lm111 in promoting the adhesion of 4-1BB-expressing cells (Figure 5C). The adhesion of HEK293^m4−1BB^ cells was specific since no adhesion of wild-type HEK293 cells to BSA- and hCEA-coated wells was observed (Figure 5A). Furthermore, wells coated with BSA- and hCEA, preincubated with 1D8^N18^ did not support any significant adhesion of 4-1BB-positive and 4-1BB-negative cells (Figures 5A,B).

To determine whether 1D8^N/C^CEA.1 retained the baseline costimulatory capacity observed for 1D8^N18^ (10), and whether this was improved by the interaction to hCEA. Mouse CD8a^+^ T cells were stimulated with plate-bound anti-CD3 mAb and the costimulatory antibodies 1D8 IgG, 1D8^N18^ and 1D8^N/C^CEA.1 in solution, in the presence or absence of immobilized hCEA. A rat IgG_2a_ and the CEA-specific MFE-23 scFv-based N-terminal trimerbody MFE-23^N18^ (10) were used as controls. In the absence of hCEA the 1D8^N/C^CEA.1 showed a costimulatory effect similar to 1D8^N18^, but in the presence of immobilized hCEA T cell proliferation was significantly increased (*P* = 0.003), whereas IFNγ secretion showed a trend to increase, without statistical significance (Figures 5D,E). In the absence of concomitant anti-CD3 mAb-stimulation, the 1D8^N/C^CEA.1 did not costimulate T cells, even in the presence of immobilized hCEA (Supplementary Figure 1).

## Discussion

Enhancing 4–1BB costimulation by systemic administration of agonistic mAbs is a viable therapeutic strategy, hampered by off-tumor toxicities associated with FcγR interactions (7, 10, 15). Arguably, reducing the toxicity of immune potentiating antibodies without compromising their antitumor activity is paramount to fully exploiting their clinical potential (16). We have previously shown that an EGFR-targeted 4-1BB-agonistic trimerbody is efficiently targeted to EGFR-expressing tumors *in vivo* and exhibits antitumor activity similar to IgG-based 4-1BB-agonistic mAbs, with no evidence of toxicity (10).

Given the broad expression profile of EGFR, a CEA-targeted 4-1BB-agonistic trimerbody was generated by fusing the anti-human CEA single-domain antibody CEA.1 (13) to the C-terminus of the 4-1BB-agonistic scFv-based trimerbody 1D8^N5^ (10) through a 17-residue linker. The trimerbody 1D8^N/C^CEA.1 was expressed by gene-modified human cells as soluble protein and was very efficient at recognizing antigens. Biolayer interferometry analysis revealed that the 1D8^N/C^CEA.1 trimerbody was capable of binding concurrently to 4-1BB and CEA, indicating that each domain independently binds to its cognate antigen. SEC-MALS showed that the 1D8^N/C^CEA.1 primarily formed the expected trimeric structure. These data suggest that despite the different configuration, with shorter linkers on both ends of the collagen XVIII homotrimerization domain, the overall structure and biophysical properties of the molecule were not modified, suggesting that the 1D8^N/C^CEA.1 may have a structure similar to the one observed for the 1D8^N/C^EGa1, with the six binding domains in an extended hexagonal conformation around the homotrimerization domain (10). These results also show that the trimerbody format has an enormous structural plasticity and could generate molecules fully adapted to different pathological contexts.

CEA is a non-internalizing antigen (17), which may be advantageous compared to other antigens that are rapidly internalized after antibody binding (18), since the CEA targeted 4–1BB agonistic trimerbody may be available to bind to T cell 4-1BB for a prolonged period after binding to tumor cell CEA. Furthermore, in normal gastrointestinal tract the CEA is expressed at the apical surface of glandular epithelial cells, where it is poorly accessible to systemically administered antibodies due to tight junctions (11, 19), which might reduce potential on-target/off tumor toxicity.

Importantly, 1D8^N/C^CEA.1 recognized cell surface expressed 4-1BB as efficiently as the anti-4-1BB mAb 1D8, and it was very specific at inducing adhesion of 4-1BB-expressing cells on plastic-bound CEA, but not on plastic-bound BSA. Appropriate stimulation by the members of the TNFRSF involves oligomerization of homotrimer receptors into higher-order complexes at the immunological synapse (3), and accordingly, the potency of the CEA-targeted 1D8^N/C^CEA.1 trimerbody was superior to first-generation anti-4-1BB mAbs. The CEA-specific 4-1BB-agonistic trimerbody demonstrated better costimulation of CD8a+ T cells in the presence of immobilized hCEA. These results support the 1D8^N/C^CEA.1 trimerbody-induced formation of clustered 4-1BB signaling complexes at the contact interface between T cells and the CEA-expressing cells.

Trimerbodies correspond to a new class of therapeutic agents that offer important advantages over canonical mAbs to manipulate the immune system for therapeutic purposes (20–23), including reduced toxicity (10). Tumor-targeted 4-1BB-agonistic trimerbodies offer additional advantages compared to conventional IgG-based antibodies in terms of biodistribution, tumor localization, and therapeutic index. In fact tumor-specific trimerbodies have been employed for molecular imaging of cancer with modalities such as nuclear (24) and optical imaging (21, 25). We consider that radiolabeled trimerbodies will have potential as theranostic tools in cancer therapy, to improve personalized treatment based on the molecular characteristics of cancer cells and in the intratumoral distribution of immune cell populations, such as 4-1BB positive T cells. Additional advantages of tumor-targeted trimerbodies including the simultaneous targeting of different receptors to exert synergistic or additive effects for the promotion of protective antitumor immunity. For example, treatment with anti-4-1BB and anti-PD-L1 mAbs (26). Yet the complexity of generating and access to multiple clinical-grade mAbs, often manufactured by different companies, essentially precludes their combined exploration in clinical trials.

In summary, our results confirm the value of tumor-targeted 4-1BB agonistic trimerbodies as an alternative to canonical immunomodulatory antibodies in order to realize the therapeutic potential of 4-1BB costimulation.

## Data Availability

The datasets generated for this study are available on request to the corresponding author.

## Ethics Statement

Animal protocols were approved by the Ethic Committee of Animal Experimentation of the Instituto Investigación Sanitaria Puerta de Hierro-Segovia de Arana (Hospital Universitario Puerta de Hierro Majadahonda, Madrid, Spain). Procedures were additionally approved by the Animal Welfare Division of the Environmental Affairs Council of the Government of Madrid (66/14).

## Author Contributions

LÁ-V conceived and supervised the study. KMi, SH, MC, NM, KMø, and SL designed and performed most of the experiments. KMi, SH, MC, NM, SL, AA-M, FB, and LÁ-V analyzed and discussed the data. KMi, SH, MC, LÁ-V wrote the manuscript. All authors edited the manuscript.

### Conflict of Interest Statement

MC is a current employee, and LÁ-V is a co-founder of Leadartis. The remaining authors declare that the research was conducted in the absence of any commercial or financial relationships that could be construed as a potential conflict of interest.

## References

[1] Cabo M Offringa R Zitvogel L Kroemer G Muntasell A Galluzzi L. Trial watch: immunostimulatory monoclonal antibodies for oncological indications. Oncoimmunology. (2017) 6:e1371896. 10.1080/2162402X.2017.1371896 29209572 PMC5706611

[2] Wei SC Duffy CR Allison JP. Fundamental mechanisms of immune checkpoint blockade therapy. Cancer Discov. (2018) 8:1069–86. 10.1158/2159-8290.CD-18-0367 30115704

[3] Chester C Sanmamed MF Wang J Melero I. Immunotherapy targeting 4-1BB: mechanistic rationale, clinical results, and future strategies. Blood. (2018) 131:49–57. 10.1182/blood-2017-06-741041 29118009

[4] Sanchez-Paulete AR Labiano S Rodriguez-Ruiz ME Azpilikueta A Etxeberria I Bolanos E. Deciphering CD137 (4-1BB) signaling in T-cell costimulation for translation into successful cancer immunotherapy. Eur J Immunol. (2016) 46:513–22. 10.1002/eji.201445388 26773716

[5] Guedan S Ruella M June CH. Emerging cellular therapies for cancer. Annu Rev Immunol. (2018) 37:145–71. 10.1146/annurev-immunol-042718-041407 30526160 PMC7399614

[6] Melero I Shuford WW Newby SA Aruffo A Ledbetter JA Hellstrom KE. Monoclonal antibodies against the 4-1BB T-cell activation molecule eradicate established tumors. Nat Med. (1997) 3:682–5 10.1038/nm0697-682 9176498

[7] Melero I Hirschhorn-Cymerman D Morales-Kastresana A Sanmamed MF Wolchok JD. Agonist antibodies to TNFR molecules that costimulate T and NK cells. Clin Cancer. (2013) 19:1044–53. 10.1158/1078-0432.CCR-12-2065 23460535 PMC4397897

[8] Fellermeier S Beha N Meyer JE Ring S Bader S Kontermann RE. Advancing targeted co-stimulation with antibody-fusion proteins by introducing TNF superfamily members in a single-chain format. Oncoimmunology. (2016) 5:e1238540. 10.1080/2162402X.2016.1238540 27999756 PMC5139625

[9] Pastor F Kolonias D McNamara JO Gilboa E. Targeting 4-1BB costimulation to disseminated tumor lesions with bi-specific oligonucleotide aptamers. Mol Ther. (2011) 19:1878–86. 10.1038/mt.2011.145 21829171 PMC3188744

[10] Compte M Harwood SL Munoz IG Navarro R Zonca M Perez-Chacon G. A tumor-targeted trimeric 4-1BB-agonistic antibody induces potent anti-tumor immunity without systemic toxicity. Nat Commun. (2018) 9:4809. 10.1038/s41467-018-07195-w 30442944 PMC6237851

[11] Hammarstrom S. The carcinoembryonic antigen (CEA) family: structures, suggested functions and expression in normal and malignant tissues. Semin Cancer Biol. (1999) 9:67–81. 10.1006/scbi.1998.0119 10202129

[12] Martinez-Forero I Azpilikueta A Bolanos-Mateo E Nistal-Villan E Palazon A Teijeira A. T cell costimulation with anti-CD137 monoclonal antibodies is mediated by K63-polyubiquitin-dependent signals from endosomes. J Immunol. (2013) 190:6694–706. 10.4049/jimmunol.1203010 23690480

[13] Cortez-Retamozo V Backmann N Senter PD Wernery U De BP Muyldermans S. Efficient cancer therapy with a nanobody-based conjugate. Cancer Res. (2004) 64:2853–7. 10.1158/0008-5472.CAN-03-3935 15087403

[14] Álvarez-Cienfuegos A Nunez-Prado N Compte M Cuesta AM Blanco-Toribio A Harwood SL. Intramolecular trimerization, a novel strategy for making multispecific antibodies with controlled orientation of the antigen binding domains. Sci Rep. (2016) 6:28643. 10.1038/srep28643 27345490 PMC4921811

[15] Sanmamed MF Pastor F Rodriguez A Perez-Gracia JL Rodriguez-Ruiz ME Jure-Kunkel M. Agonists of co-stimulation in cancer immunotherapy directed against CD137, OX40, GITR, CD27, CD28, and ICOS. Semin Oncol. (2015) 42:640–55. 10.1053/j.seminoncol.2015.05.014 26320067

[16] Álvarez-Vallina L 4-1BB-mediated cancer immunotherapy:'mission impossible'for non-engineered IgGs? Precis Cancer Med. (2019) 2:1 10.21037/pcm.2019.01.01

[17] Schmidt MM Thurber GM Wittrup KD. Kinetics of anti-carcinoembryonic antigen antibody internalization: effects of affinity, bivalency, and stability. Cancer Immunol Immunother. (2008) 57:1879–90. 10.1007/s00262-008-0518-1 18408925 PMC2840397

[18] Liu Z Yu Z He W Ma S Sun L Wang F. *In-vitro* internalization and *in-vivo* tumor uptake of anti-EGFR monoclonal antibody LA22 in A549 lung cancer cells and animal model. Cancer Biother Radiopharm. (2009) 24:15–24. 10.1089/cbr.2008.0537 19216631

[19] Bacac M Fauti T Sam J Colombetti S Weinzierl T Ouaret D. A novel carcinoembryonic antigen T-cell bispecific antibody (CEA TCB) for the treatment of solid tumors. Clin Cancer. (2016) 22:3286–97. 10.1158/1078-0432.CCR-15-1696 26861458

[20] Blanco-Toribio A Sainz-Pastor N varez-Cienfuegos A Merino N Cuesta AM Sanchez-Martin D. Generation and characterization of monospecific and bispecific hexavalent trimerbodies. MAbs. (2013) 5:70–9. 10.4161/mabs.22698 23221741 PMC3564888

[21] Cuesta AM Sanchez-Martin D Sanz L Bonet J Compte M Kremer L. *In vivo* tumor targeting and imaging with engineered trivalent antibody fragments containing collagen-derived sequences. PLoS ONE. (2009) 4:e5381. 10.1371/journal.pone.0005381 19401768 PMC2670539

[22] Cuesta AM Sainz-Pastor N Bonet J Oliva B varez-Vallina L. Multivalent antibodies: when design surpasses evolution. Trends Biotechnol. (2010) 28:355–62. 10.1016/j.tibtech.2010.03.007 20447706

[23] Cuesta AM Sanchez-Martin D Blanco-Toribio A Villate M Enciso-Alvarez K varez-Cienfuegos A. Improved stability of multivalent antibodies containing the human collagen XV trimerization domain. MAbs. (2012) 4:226–32. 10.4161/mabs.4.2.19140 22453098 PMC3361658

[24] Rios X Compte M Gomez-Vallejo V Cossio U Baz Z Morcillo MA. Immuno-PET imaging and pharmacokinetics of an Anti-CEA scFv-based trimerbody and its monomeric counterpart in human gastric carcinoma-bearing mice. Mol Pharm. (2019) 16:1025–35. 10.1021/acs.molpharmaceut.8b01006 30726099

[25] Sanchez-Martin D Cuesta AM Fogal V Ruoslahti E varez-Vallina L. The multicompartmental p32/gClqR as a new target for antibody-based tumor targeting strategies. J Biol Chem. (2011) 286:5197–203. 10.1074/jbc.M110.161927 21156793 PMC3037632

[26] Hirano F Kaneko K Tamura H Dong H Wang S Ichikawa M. Blockade of B7-H1 and PD-1 by monoclonal antibodies potentiates cancer therapeutic immunity. Cancer Res. (2005) 65:1089–96. 15705911

