# Supplementary material for: Carcinoembryonic Antigen (CEA)-Specific 4-1BB-Costimulation Induced by CEA-Targeted 4-1BB-Agonistic Trimerbodies
Source: Front Immunol. 2019 Jul 31;10:1791. doi: 10.3389/fimmu.2019.01791 (PMC6685135; doi:10.3389/fimmu.2019.01791)
Supplement: Supplementary file 1 [file Data_Sheet_1.PDF]

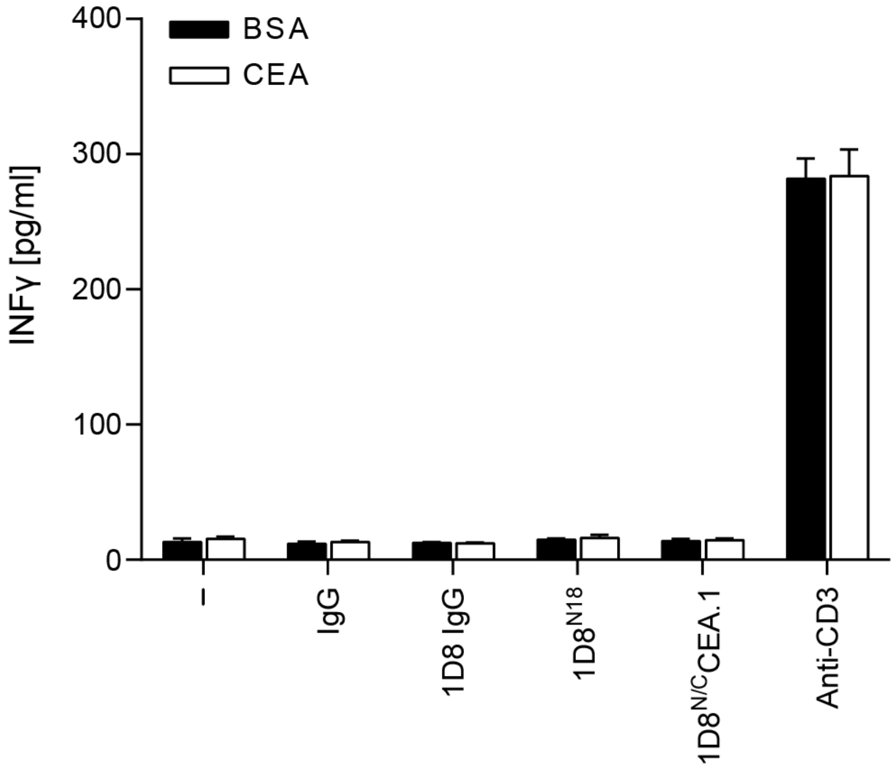

Supplementary Figure 1. Secretion of interferon-γ by mouse CD8+ T cells in the presence of soluble antibodies and immobilized antigens. Mouse CD8a+ T cells were plated with immobilized hCEA or BSA in the presence of rat IgG, 1D8 IgG, $1D8^{N18}$, $1D8^{N/C}$CEA.1, $MFE23^{N18}$ or anti-CD3 mAb, and IFN-γ secretion was determined after 48 hours. All the results are representative of one of three independent experiments. Data are mean ± SD (*n* = 3).
